# A Curious Case of Proptosis and Intracranial Calcifications Caused by a Vein of Galen Aneurysmal Malformation

**DOI:** 10.7759/cureus.47453

**Published:** 2023-10-22

**Authors:** Sandra C M, Breman A Peethambar

**Affiliations:** 1 Diagnostic Radiology, MES (Muslim Educational Society) Medical College, Perinthalmanna, IND; 2 Medicine, Madras Medical College, Chennai, IND

**Keywords:** digital subtraction angiography, cardiac failure, intracranial calcifications, proptosis, vein of galen

## Abstract

Vein of Galen aneurysmal malformation (VGAM) is a rare, congenital, intracerebral arteriovenous malformation with a poor prognosis. This disorder commonly presents during the neonatal period and rarely in infancy and childhood. Reported here is a case of VGAM in a three-month-old female baby who presented with proptosis and intracranial calcifications, which are rare presentations of this rare entity. The diagnosis was confirmed by magnetic resonance imaging (MRI).

## Introduction

Vein of Galen aneurysmal malformation (VGAM) is a rare congenital arteriovenous malformation caused by fistulous connections between cerebral arteries and cerebral veins leading to an abnormally persistent and dilated median prosencephalic vein of Markowski, which serves as an embryologic precursor to the vein of Galen. This occurs between the sixth and eleventh week of gestation and has a male predominance [1]. Symptoms can vary based on age and anatomy with high output cardiac failure, irreversible brain damage, and macrocephaly being usual presenting signs of VGAM. Presented here is a case of VGAM with two rare presentations.

## Case presentation

An 86-day-old female baby presented to the ophthalmology department with a two-week history of swelling and redness of the left eye and two episodes of generalized tonic-clonic seizures. There was no history of trauma or eye discharge. She was born at 39 weeks of gestational age to a 27-year-old G1P1 via spontaneous vaginal delivery and weighed 2.7 kg at birth. Her antenatal, family, and medical history were unremarkable.

General physical examination revealed a temperature of 98.6⁰F, heart rate of 130 beats per minute, blood pressure of 78/41 mmHg, respiratory rate of 32 per minute, and oxygen saturation of 100% on room air. The baby had pallor, a capillary refill time of less than 3 seconds, and weighed 4.1 kg. The shape of the head was normal. Left eye proptosis and dilated episcleral vessels were noted. A neurological examination revealed a drowsy child. Power, tone, bulk, and reflexes were within normal limits. There were no signs of meningeal irritation. Respiratory, cardiovascular, and gastrointestinal system examinations were unremarkable.

**Figure 1:**
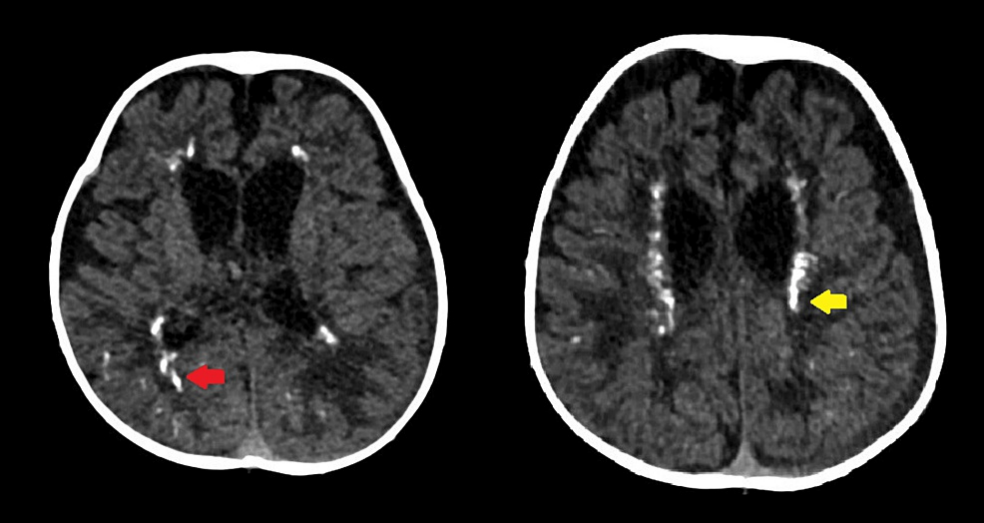
Axial CT images of the brain showed linear hyperdensities in the subcortical white matter of bilateral parietal lobes (red arrow) and periventricular regions (yellow arrow) suggestive of intracranial calcifications. Ventricles appeared mildly dilated. Increased subarachnoid space was noted in bilateral frontoparietal regions suggestive of benign enlargement of the subarachnoid space in infancy.

Cerebral computed tomography (CT) and MRI scans were requested for further characterization. Noncontrast CT of the brain showed intracranial calcifications in the subcortical white matter of the parietal lobes and periventricular regions of the frontal lobes bilaterally. Ventricles appeared mildly dilated. Increased subarachnoid space was noted in bilateral frontoparietal regions suggestive of benign enlargement of subarachnoid space in infancy (Figure 1).

A TORCH (Toxoplasmosis, Rubella, Cytomegalovirus, and Herpes Simplex) panel was sent because of intracranial calcifications, which turned out to be negative.

**Figure 2:**
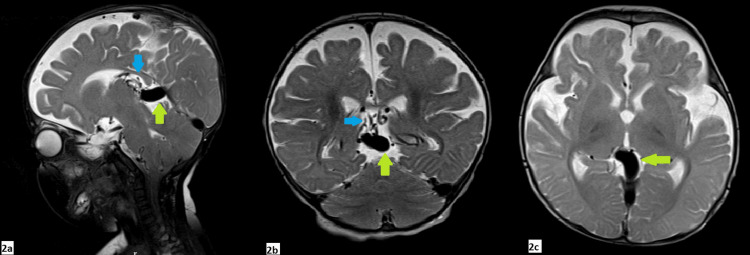
Sagittal (2a), coronal (2b), and axial (2c) 1.5T MR images of the abdomen showed a characteristic appearance of VGAM with a large flow void in the midline, posterior to third ventricle representing enlarged median prosencephalic vein of Markowski of maximum diameter 7 mm (green arrows). Arterial feeders were seen along the anterior wall of the vein (blue arrows).

**Figure 3:**
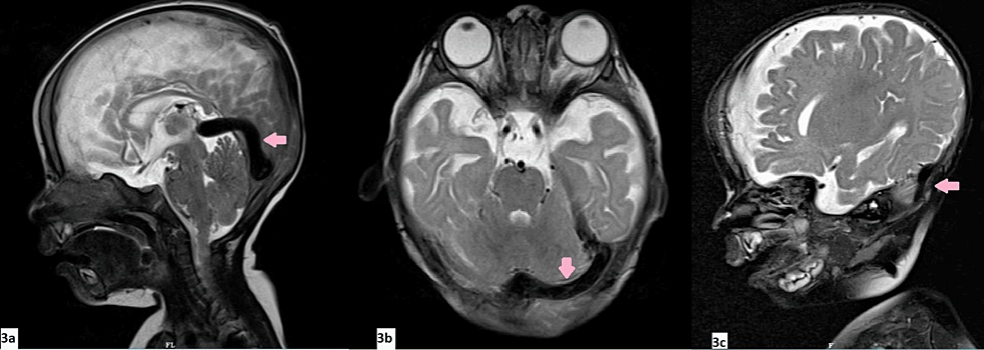
Sagittal (3a and 3c) and axial (3b) T2-weighted MR images showed dilatation of the straight sinus (3a), left transverse sinus (3b), and left sigmoid sinus (3c)

**Figure 4:**
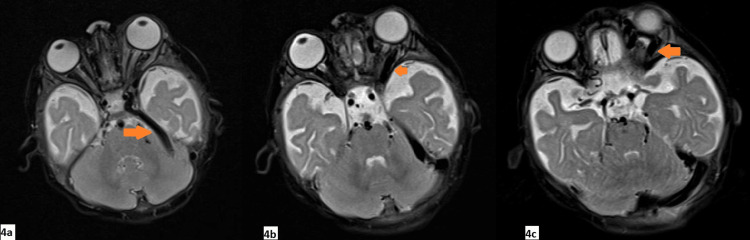
Axial (4a, 4b, and 4c) T2-weighted MR images showed dilatation of the left superior petrosal sinus (4a) and left superior ophthalmic vein (4b and 4c)

MR images showed a characteristic appearance of VGAM with a large flow void in the midline, posterior to third ventricle extending to the straight, left transverse, left sigmoid, left superior petrosal sinuses, and left cavernous sinuses and the left superior ophthalmic vein. Numerous arterial feeders were seen along the anterior wall of the VGAM (Figures 2-4).

**Figure 5:**
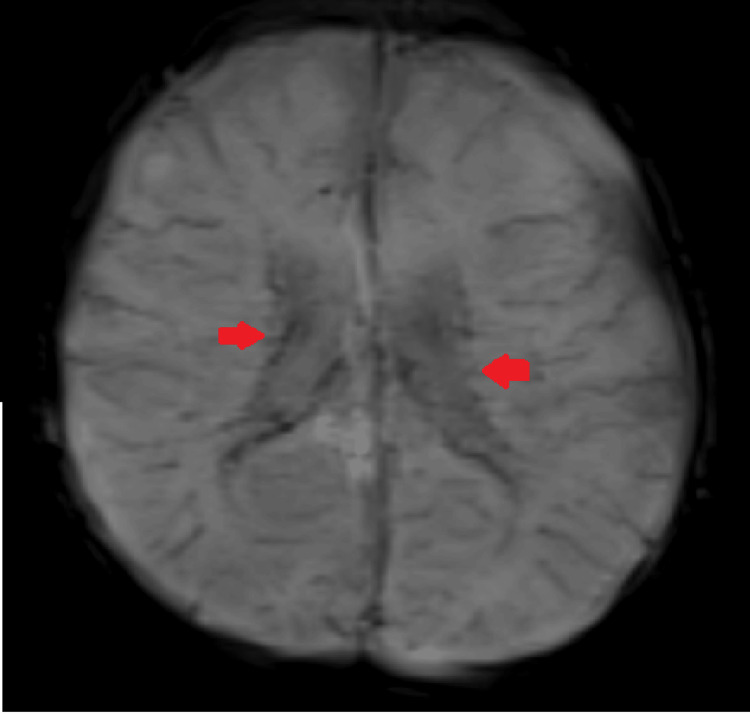
Susceptibility-weighted image (SWI) showed multiple, tiny blooming areas in periventricular regions, which concurred with the calcifications previously seen on CT

This patient subsequently underwent endovascular embolization.

## Discussion

VGAM is a rare form of embryonic arteriovenous shunt, which consists of multiple feeding arteries draining into the median vein of the prosencephalon, which fails to regress between 6 and 11 weeks of gestation [2]. It accounts for less than 1-2% of all intracranial vascular malformations. VGAMs commonly present after birth with cardiac failure, failure to thrive, or macrocephaly. Very few case reports with VGAM presenting as proptosis have been reported in the literature to date [3,4]. Based on the location and nature of the shunt, VGAMs are divided into two main subtypes, choroidal and mural [1]. The choroidal type consists of communication between bilateral arterial feeders from choroidal arteries, thalamic perforator arteries, pericallosal arteries, and the anterior segment of the median vein of the prosencephalon [5]. The large volume of shunt and aggressive volume overload results in cardiac failure, cerebral ischemia, and encephalomalacia [6]. The mural type of VGAM consists of a single arteriovenous fistula between unilateral or bilateral collicular and/or posterior choroidal arteries and the inferolateral wall of the median vein of Markowski. This type presents in infancy or childhood with failure to thrive, macrocephaly, or heart failure [5].

VGAMs are sometimes detected in late antenatal and neonatal transfontanellar ultrasound scans. The dilated median prosencephalic vein (MPV) appears as an anechoic structure in the midline posteriorly and demonstrates prominent flow on Doppler examination. However, diagnosis on an antenatal scan is not a common occurrence due to the low pressures in the venous system before birth [7]. Plain radiography of the skull often reveals a curvilinear peripheral rim of calcification within the wall of the aneurysmal sac [8].

Contrast-enhanced CT shows a well-defined, midline, intensely enhancing lesion with the cistern of velum interpositum. This is often associated with dilatation of ventricles, periventricular white matter hypodensities and calcifications, and diffuse cerebral atrophy. The parenchymal calcifications can be attributed to chronic venous hypertension and ischemia leading to the secondary development of dystrophic subcortical white matter calcifications [8]. MRI augmented by MR angiography and MR venography reveals the dilated feeding and draining vessels in greater detail. The aneurysmal dilatation and its associated arteries and veins appear as flow voids on T1 and T2. MRI can demonstrate the location of the fistula, the presence of any nidus, and thrombosis of the venous sac along with a better description of anatomy before treatment.

Digital subtraction angiography is diagnostic and therapeutic and remains the gold standard for the evaluation of VGAMs. It allows us to precisely demonstrate the angioarchitecture of the malformation and catheterize feeders supplying the fistula. Endovascular embolization has now become the optimal approach for VGAM with a good clinical outcome and acceptable mortality. The prognosis depends on the size of the shunt and the presence of cardiac failure in utero [2,9].

## Conclusions

VGAMs commonly present with high-output cardiac failure in the neonatal period. However, they should be considered as a differential diagnosis in children presenting to the ophthalmology department with proptosis. Although rare, radiologists should include VGAM in the differential diagnosis of intracranial calcifications in children. Untreated VGAMs are almost always fatal. Hence, it is of utmost importance to diagnose this challenging condition as early as possible to initiate proper management.
